# The Conformational Plasticity Vista of PDZ Domains

**DOI:** 10.3390/life10080123

**Published:** 2020-07-27

**Authors:** Javier Murciano-Calles

**Affiliations:** Departamento de Química Física, Unidad de Excelencia de Química Aplicada a Biomedicina y Medioambiente, Facultad de Ciencias, Universidad de Granada, 18071 Granada, Spain; jmurciano@ugr.es

**Keywords:** PDZ domains, PSD95, protein folding, ligand binding, allostery, post-translational modifications

## Abstract

The PDZ domain (PSD95-Discs large-ZO1) is a widespread modular domain present in the living organisms. A prevalent function in the PDZ family is to serve as scaffolding and adaptor proteins connecting multiple partners in signaling pathways. An explanation of the flexible functionality in this domain family, based just on a static perspective of the structure–activity relationship, might fall short. More dynamic and conformational aspects in the protein fold can be the reasons for such functionality. Folding studies indeed showed an ample and malleable folding landscape for PDZ domains where multiple intermediate states were experimentally detected. Allosteric phenomena that resemble energetic coupling between residues have also been found in PDZ domains. Additionally, several PDZ domains are modulated by post-translational modifications, which introduce conformational switches that affect binding. Altogether, the ability to connect diverse partners might arise from the intrinsic plasticity of the PDZ fold.

## 1. Introduction

Protein folding and binding are intertwined aspects of protein function. Traditionally, and still today, the relation between protein structure and function is one of the cornerstones of protein science research. Binding is essentially related to almost the entirety of protein functionality: signaling, catalysis, transport, etc. Thus, the process of reaching the functional structure, i.e., folding, is objectively a basis for protein binding. Nevertheless, a direct relationship between folding and binding is not as easy to find as one might expect in principle. Moreover, the discovery of the intrinsically disordered proteins (IDPs) has launched a new challenge since the folding process is triggered by a binding event [1].

Currently, a widely accepted view of protein folding is that of the energy landscape [2,3]. In such a view, ensembles of myriads of conformational states are dynamically interchanging one into another, as is depicted, for instance, in molecular dynamics simulations. Some of those conformations are capable of binding the protein target(s). Moreover, there is generally a conformation stabilization upon binding. In this way, folding is connected to binding, and both aspects have even been considered as the two sides of the same coin [4]. 

In this interesting link between protein folding, binding, and function, PDZ domains (whose name derives from the original proteins in which they were discovered: PSD95-Discs large-ZO1) has been a thoroughly studied example. PDZ domains are protein modules highly abundant in nature. One of the main functions of this domain family is to work as hub proteins that interconnect multiple partners in signaling (see, for example, [5,6] for PDZ as scaffold proteins interconnecting multiple processes). It is striking that a single protein domain can serve as a scaffold to diverse and numerous targets and mediate signaling networks. A hypothetical reason for PDZ versatility may be found in a wide range of conformational features that confer high plasticity to the domain. Indeed, plasticity has been a peculiarity that has been associated with PDZ domains [7,8,9], mainly in regards to both the detection of different-in-nature intermediates in their folding and allostery in their binding. This review gives prominence to the vast number of studies of PDZ folding and extensive knowledge of protein folding landscapes derived from it. Then, an analysis more centered on a paradigmatic PDZ domain studied up to date, the third PDZ domain of PSD95 (Post-synaptic density, 95 kDa) protein (PSD95-PDZ3), sheds light on other different aspects including misfolding, allostery, and how post-translational modifications alter function and binding by conformational changes. The next section conveys the case of a PDZ domain direct correlation between folding and binding. Finally, plasticity in PDZ targets is also briefly remarked. 

## 2. PDZ Folding and Implications in the Folding Mechanism Theories

There are extensive works and bibliographies on PDZ folding (and binding) carried out by Gianni and Jemth research groups. Several reviews on their research, from different points of view, can be consulted: the earlier studies on folding and binding in PDZ domains [10], the molecular details and mechanisms on ligand binding in PDZ domains and implications as drug targets [11], or an insightful reflection on the allosteric phenomenon in the PDZ domain [12], among others. Here, a comprehensive collection of PDZ folding studies, including the very last articles, is summarized in Table 1. Two other references involve PDZ folding as well [13,14], although they are computational studies based on the experimental data obtained in the references contained in Table 1. 

The work of Gianni and Jemth in PDZ folding has been founded mostly on kinetic experiments. Stopped-flow measurements allowed the construction of Chevron plots that were subsequently analyzed by different mathematical equations deducted by a theoretical model. A basic principle in fitting data is to accept the simplest model that can explain the results. Thus, the two-state model (comprising native and unfolded states) is commonly considered first. If the fitting is not adequate, then other more complex models, including intermediates, are investigated. In addition, to decipher the kinetic folding model of PDZ domains, the transition states have been characterized for several of them through φ-value analysis (see [31,32,33] for the fundamentals of the methodology). Since a transition state never accumulates, any information has to be inferred indirectly. Interaction patterns are delineated by mutation of selected side chains and measuring the kinetics and stability of the mutants. The φ-value is the result of the division of the activation free energy change and the stability change in the mutant with respect to the native state. A posterior use of the φ-values as restraints in molecular dynamics simulations allows the depiction of the transition state structure.

What can we learn from the folding mechanism of seven different PDZ domains, together with several circularly permuted variants, other types of mutants, and even a spliced form of one of them? The answer is clear: a deeper understanding of the protein folding theories. In the early sixties of the last century, Chris Anfinsen posed the folding problem [34]: How can a protein fold? What is the mechanism of the folding process? The two-state behavior was seen to not have intermediates. Just a few years later, Cyrus Levinthal estimated that the required time for folding by just random exploration is even longer than the life of the universe, concluding that folding intermediates and pathways should be present [35]. Two approaches tried to explain the inquiries. The first model considered folding as a fast formation of nuclei containing secondary structure segments, with a subsequent collision between those nuclei, in the so-called diffusion–collision model [36]. The second model, nucleation–condensation, is the synchronized formation of both the secondary and tertiary structures from a leading nucleus that is somewhat a deformed version of the native conformation. Then, upon condensation, the nucleus adopts the native structure [37]. 

What the work on PDZ domains has yielded is the presence of proteins that behave according to those two models. The mutational study of the kinetics of PTP-BL PDZ2 showed an intermediate and two transition states, TS1 and TS2. TS1 was shown to have just a few native-like contacts, which is in accord with the nucleation–condensation mechanism, whereas TS2 showed more consistent elements of a secondary structure already formed [16], as it is postulated in the diffusion–collision model. Hence, the PDZ domain case confirmed a unifying mechanism that had previously been proposed in which both models are included. This unifying model suggests that, depending on the intrinsic stability of the secondary structure elements [38,39], one of the two defined models would be manifested. Interestingly, for PTP-BL PDZ2, both extreme models define its folding mechanism. 

A broadly accepted view of protein folding nowadays is that of the energy landscape theory [3]. The idea is that proteins are dynamic ensembles of conformations, each one associated with an energy potential delineating an energy landscape. The shape of the landscape is rugged, containing hills and valleys with small or high barriers, and funneled, leading to the most stable or native conformation. According to this view, the plausibility of the two models in the same protein is congruent, since the energy landscape may be vast and folding can be reached through winding roads in the search for minimum energies. Moreover, if the energy differences between the alternative routes are not high, protein folding might occur differently depending on conditions. This means that the energy landscape is tunable, depending mainly and foremost on the sequence protein (including mutations, altered topologies, truncations…) and also on experimental conditions. Again, the exhaustive work on PDZ domains folding gives light on the heterogeneity of the landscapes and how protein folding can be led and/or pass through different routes. 

The most interesting case in this regard is the already mentioned PTP-BL PDZ2 domain. The described intermediate was shown to be on-pathway and productive towards the native state as it was revealed through kinetic analysis of protein stabilization upon ligand binding [17]. Importantly, a circularly permuted variant showed another intermediate that was reached by alternative folding nuclei different from that of the wild-type [19]. Moreover, a naturally occurring spliced form showed that the folding nuclei towards the intermediate were again different. However, the second transition state (from the intermediate to the native state) was similar to that found for the wild-type. Hence, TS1 has been revealed to be more malleable than TS2 [21]. As a consequence, altering the topology or the sequence of PTP-BL PDZ2 produces a TS2 with more similar structures between the variants. The contrary happens with TS1; the structures differ between the variants. This fact is in accord with the view that the native topology drives the folding mechanism [40]. In addition, it nicely reflects the funneled shape of the landscape, which becomes narrower (keeping the analogy of the funnel) when it gets closer to the very bottom and end of it. 

Another clue for portraying an energy landscape in the case of the D1pPDZ domain, a naturally circularly permuted PDZ domain. An intermediate was also detected in the folding route, although rather different from those previously detected. In a set of PDZ domains, an on-pathway productive intermediate was shared [22], but in D1pPDZ, the intermediate was off-pathway and, interestingly, misfolded. Indeed, the structural characterization of the intermediate highlighted the alternative folding route that takes place in D1Ppdz [29], where the residues around the binding pocket are the main responsibility of the misfolded intermediate. Seemingly, nature preferred to conserve the responsible residues for binding to preserve functionality over the risk of incorrect folding.

**Figure 1:**
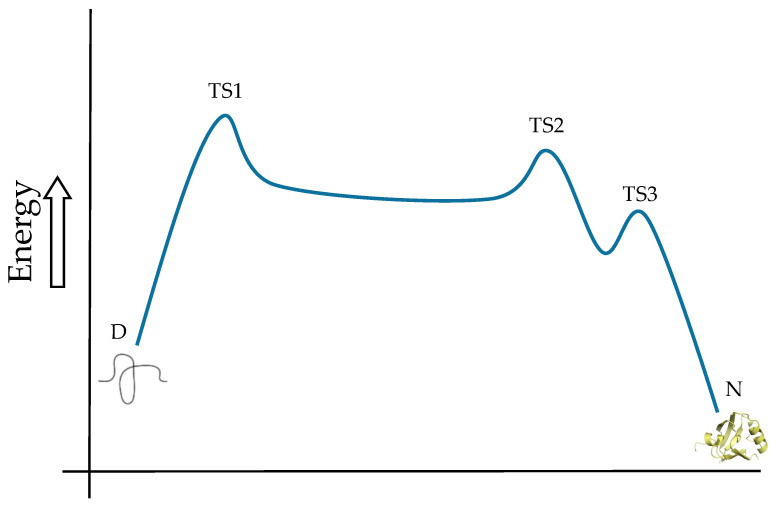
Schematic energy diagram showing the folding pathway of a PDZ domain. Three transition states (TS) between two intermediates are present in the course from the denatured state (D) to the native state (N). Transition states closer to the native state comprise more native-like interactions reinforcing the concept of native topology driving the folding mechanism. The PDZ structure selected in the figure as the native state is one of the crystallographic structures of PSD95-PDZ3 (PDB code 3I4W), one of the most studied PDZ domains (see Section 3).

The final meaningful example of PDZ domains and the energy landscape of protein folding is the SAP97 PDZ2 domain. In addition to the previous examples, this PDZ domain shows again an intermediate, with the particularity that it may be on- or off-pathway. The ruggedness of the landscape is, therefore, glimpsed since results showed that when changing experimental conditions, the folding mechanism was re-routed [7]. Additionally, the re-analysis of the whole data on the kinetics of all the PDZ domains studied (see Table 1) led to another plausible scenario, with two intermediates and three transition states [23]. Figure 1 shows the postulated scheme of the folding route common to the PDZ fold, according to [23]. Conclusively, the landscape is rugged, and the routes can be diverse and altered by mutations, topology, and factual conditions. 

Undoubtedly, the accumulated information offered by the vast work on PDZ domain folding enriches and enlightens the theories about protein folding. The experimental data also provide evidences about the energy landscapes of the PDZ fold, substantiating the plasticity of this domain family.

## 3. The Third PDZ Domain of PSD95: Folding Intermediates, Allostery, and Post-Translational Modifications 

PSD95 is a post-synaptic density protein that interconnects multiple processes [5,41]. It is considered as a hub protein [42], which is part of the so-called scale-free biological networks [43,44], whose main feature is the presence of nodes that connect numerous partners. A central question about hub proteins is how they can interact with such a number and different kinds of targets. Hub proteins may be either modular proteins or IDPs [45,46]. PSD95 is one of the first kind, a modular protein comprising five domains: three PDZ, one SH3, and one Guanylate Kinase. The versatility of PSD95 may originate from either the composing domains or the linkers or even an interplay of both the domains and their linkers. Considering the relevance and abundance of the PDZ domains, the interest was initially focused on PSD95 domains. Particularly, the third PDZ domain of PSD95 (PSD95-PDZ3) has been subjected to a thorough research from multiple points of view trying to decipher clues to explain its flexible functionality. 

In this section of the review, three different aspects of PSD95-PDZ3 are going to be considered: the presence of diverse intermediates in PSD95-PDZ3 folding, an allosteric phenomenon in ligand binding, and the effect of post-translational modifications on ligand binding. 

### 3.1. Folding Intermediates in the Landscape of PSD95-PDZ3

Up to three intermediates in PSD95-PDZ3 folding have been characterized by different approaches. The initial work on the folding of this domain was carried out by Feng and co-workers performing stopped-flow and native-state hydrogen exchange experiments. The authors enunciated the presence of an intermediate detected by hydrogen exchange and located after the rate-limiting step in the folding pathway [25]. The described intermediate is mostly structured native-like, with some disordered residues of the N-terminus and the α3 helix (Figure 1, Panel A). An extra β-hairpin is also disorganized, but it does not belong to the PSD95 protein structure strictly speaking (see [47,48] for clarification). 

The second described intermediate was inferred after kinetic experiments and the φ-value analysis of multiple mutants of PSD95-PDZ3 [26]. In the study, two transition states were described; one leading to the intermediate, TS1, and the other towards the native state, TS2. The corresponding structural model of TS1 showed that the initial stages of folding refer to the formation of α2 helix and β1 and β2 sheets (Figure 1, Panel B). Compared to the two transition states of PTP-BL PDZ2, TS1 from both domains differ considerably, whereas TS2 are structurally similar in both domains, which is another reminder of the fact that native topology drives folding mechanism [40]. 

The third intermediate was initially detected by differential scanning calorimetry (DSC). Calorimetric traces had two clear transitions whose maxima changed depending on protein concentration [48]. The two transitions indicate the presence of at least three states, and the observed concentration dependence revealed an association–dissociation process for the intermediate state. NMR (Nuclear magnetic resonance) and FTIR(Fourier transform infrared) spectroscopy showed that the intermediate kept the contacts around β5 (Figure 1, Panels C-1 and C-2), whereas the region around β3 is re-organized to form the seed of a misfolding route that ends in annular and fibrillar structures [48,49]. Interestingly, the misfolded intermediate is shared in other PDZ domains [50]. 

The experiments described so far always included a third alpha-helix (α3) that is not considered as a structural element in the usual PDZ fold. α3 helix in PSD95 connects the third PDZ and the SH3 domains, and it has a relevant role in function and binding (see Section 3.2 and Section 3.3). The first experiments with PSD95-PDZ3 were done with such helices, and one of the earliest PDZ structures reported included the α3 helix [51]. The removal of α3 in PSD95-PDZ3 has important consequences for the stability and misfolding events in the domain. Without the helix, the misfolded intermediate is different, and the fibrillation process is accelerated [52,53]. α3 might, therefore, protect the domain from misfolding. Nevertheless, the kinetic experiments of PSD95-PDZ3 without α3 helix showed that the folding mechanism is just marginally altered [27]. Moreover, according to the kinetic data, the folding transition state seems to have α3 unstructured. Regardless, the intermediate species detected by DSC and kinetics are different, since the latter is a high-energy species that never accumulates. 

**Figure 2:**
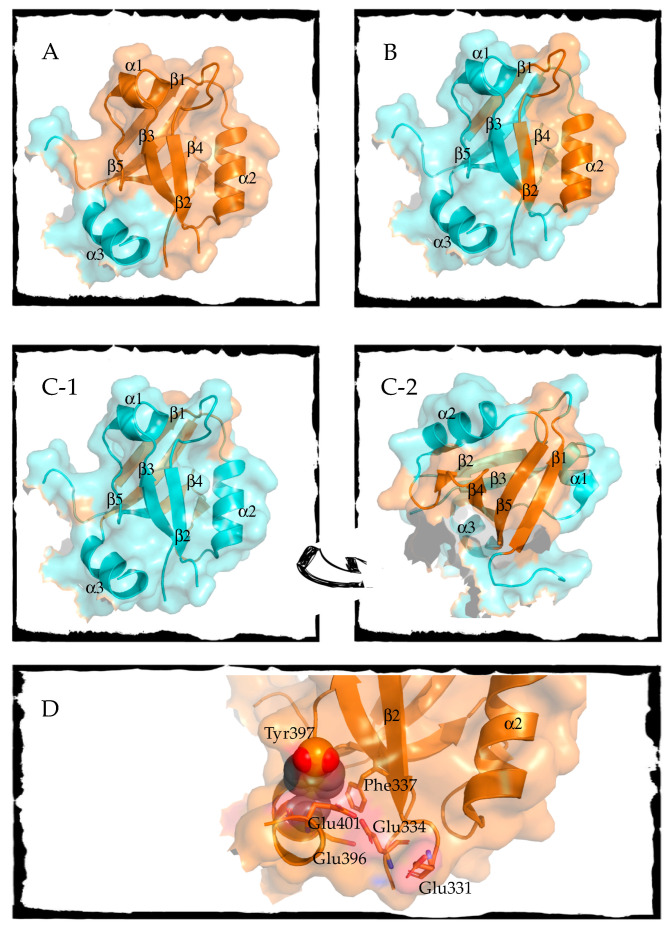
Panels A to C represent PSD95-PDZ3 structures color-coded according to the folded regions found in the experimentally detected intermediates. Cyan color corresponds to a non-organized region and orange to a natively folded region. Secondary structure elements are labeled in the figures. The panels correspond to each of the intermediates mentioned in the text: (**A**) PSD95-PDZ3 intermediate detected by native-state hydrogen exchange by Feng et al. [25]; (**B**) First transition structure (TS1) of PSD95-PDZ3 previous to the intermediate obtained by φ-values and molecular dynamics by Calosci et al. [26]; (**C**) PSD95-PDZ3 intermediate detected by differential scanning calorimetry (DSC) by Murciano-Calles et al. and characterized by FTIR (Fourier transform infrared spectroscopy) and NMR (Nuclear magnetic resonance) [48,49]; the arrow between (**C-1**) and (**C-2**) indicates a switch of the same figure to show more clearly the folded regions in orange. Panel (**D**) shows a detailed view of the location of residue Tyr397, whose phosphorylation may distort helix α3 and, therefore, alters binding in PSD95-PDZ3. A phosphate group (phosphorus in orange and oxygens in red) has been manually added to Tyr 397, which is shown in spheres to resemble the bulkiness of the side chain. Phe337 and the glutamic residues that surround Tyr397 have been shown in sticks. β2 strand and α2 helix have been labeled to note that the target proteins or peptides that interact with PSD95-PDZ3 are assembled between those two structural elements. The importance of α3 for PSD95-PDZ3 binding is highlighted in the text.

Analogously to previous cases described in Section 2, the PSD95-PDZ3 folding landscape is markedly rugged. Three different intermediates have been experimentally detected in different conditions, and they comprise a distorted native structure. Remarkably, the available information about the intermediates’ structures discloses that diverse protein regions maintain the native-like contacts in the three intermediates, as can be seen in Figure 2. Certainly, if we have been able to detect such a variety of plausible configurations, there is a high chance that in vivo, this PDZ domain is capable of adjusting its structure to adapt to different targets.

### 3.2. Allosteric Phenomena in PSD95-PDZ3 Ligand Binding

Based on evolutionary data, in 1999, Lockless and Ranganathan described energetic interactions between all the residues present in PSD95-PDZ3 through multiple sequence alignment of the whole PDZ family [54]. By mutational analysis of several variants, the authors found three regions of the PSD95-PDZ3 that were energetically coupled to His372, which had been shown to be crucial in the binding specificity (His372 forms a hydrogen bond with the hydroxyl group of a Ser or Thr residue present in the ligand). Importantly, Ranganathan and co-workers triggered the multiple allosteric analysis that has been performed on PSD95-PDZ3 and also other PDZ domains. Indeed, Ranganathan’s lab applied statistical coupled analysis on PSD95-PDZ3, showing that certain protein sectors within the domain are responsible for the allosteric control [55]. Additionally, following the same approach, they discovered that mutations on the allostery-controlling sectors can switch the domain specificity [56]. Relevant mutations for binding in PSD95-PDZ3 were also found to be distant to the binding site [9,57], which has been observed in other PDZ domains [58,59,60].

Several research groups have also studied the importance of the extra-domain α3 helix in PSD95-PDZ3 in terms of binding and allostery. Lee et al. found that the removal of α3 induces an entropy-driven loss in affinity [61]. NMR relaxation experiments showed that side-chain dynamics increased in PSD95-PDZ3 residues. In principle, the higher flexibility upon α3 removal may be due to the connecting interaction between the β2-β3 loop and α3, which makes PSD95-PDZ3 more rigid and stabilized [62,63]. Interestingly, crystallographic structures confirmed the direct relation between the conformation acquired in α3 and the configuration of the β2-β3 loop [64]. Moreover, a perturbation scanning response computational approach confirmed that the loss in affinity due to α3 removal cannot be predicted simply with the rigid crystal structures of the domain, but rather by a computational generation of multiple conformations by residue fluctuations [65]. 

Allostery has been intensely studied since Monod introduced the concept [66], and the meaning has evolved over the years (for an excellent review on the concept of allostery see [67]). Lately, allostery has also been defined as the energetic coupling of the protein residues, which is the cause of a binding alteration with changes that do not directly affect the binding pocket. Ligand binding is, therefore, influenced by protein dynamics and energetic coupling between residues in PSD95-PDZ3, referring again to its intrinsic plasticity and possibly being another way of function regulation. 

### 3.3. Post-Translational Modifications Regulate PSD95-PDZ3 Function through Conformational Perturbations

Tyr397, which belongs to α3 in PSD95-PDZ3, was found to be phosphorylated in vivo [68]. NMR and calorimetric experiments showed that the phosphorylated Tyr397 weakens ligand binding affinity and disturbs the packing of α3 [69]. Molecular dynamics simulations predicted that the inclusion of negative charges of the phosphate group may be the reason for α3 distortion [63]. In the region of α3, there are several Glu and Asp residues, and increasing the negative electrostatic potential might lead to the opening of the helix. Concretely, Panel D of Figure 2 shows four Glu residues (331, 334, 396, and 401) around the α3 region of Tyr397. The inclusion of the bulky and negatively charged phosphate group in the hydroxyl group of the side chain in Tyr397 affects the core of α3, whose electronegative potential is supplemented by the π-electron cloud of aromatic residues, such as Phe337. The prediction of helix distortion upon increasing the electrostatic potential is in accord with folding studies in which PSD-95-PDZ3 shows a two-state behavior at pH values lower than 3 [70], where Glu and Asp residues are protonated. Upon protonation of Glu and Asp at acidic pH values, the packing of the helix is increased, the equilibrium intermediate is not detected, and the folding mechanism is simpler than the three-state unfolding process previously described. The influence of the phosphorylated Tyr397 was further ratified by the binding modulation of different in vivo targets of PSD95-PDZ3 and other phosphorylation sites along the whole PSD95 protein [71].

Another plausible post-translational modification that can affect PSD95-PDZ3 is succinimide formation. Succinimide is an Asp/Asn derivative obtained after cyclization of the side chain, which often occurs in loops. A succinimide ring was observed in residue Asp332 from the β2-β3 loop in a crystal structure of PSD95-PDZ3 and confirmed by mass spectrometry of the solution of the crystal [47]. Hence, a succinimide formation, either spontaneous or induced in PSD95-PDZ3, cannot be discarded in vivo. The rigidity that confers succinimide in the β2-β3 loop was tested by mutating Asp332 to Pro, which resulted in a significant decrease in ligand binding affinity. The reduction in conformational freedom in the loop hindered the correct formation of the interactions, as was shown by molecular dynamics [63]. Regardless of the in vivo chance of succinimide formation in PSD95-PDZ3, the mutant showed that a conformational alteration clearly affects binding and, therefore, functionality. These two examples show the great importance of the structural diversification that post-translational modifications imbue in protein folding and how binding is altered as well. 

## 4. A Correlation between Binding Affinity and the Folding Mechanism in PTP-Bl Pdz2

The vast amount of work performed on PTP-BL PDZ2 also included binding studies [72,73]. The mutational studies were focused both on folding and binding events. Interestingly, the authors attempted to find a connection between their experimental data on both events. The construction of cross-correlated plots of ΔΔG upon mutation in regards to binding (kinetics constants *k_on_* and *k_off_*; and *K_D_*) and folding parameters (unfolding rate constant, *k_u_*) yielded a direct correlation between the kinetics binding constant *k_on_*, and the folding rate constant *k_u_*, together with stability. Such a correlation was not found in the other examined magnitudes. These results show a direct correlation between the formation of the nuclei that leads to the native structure and ligand binding [10]. Numerous other examples have directly linked conformational states and binding [74,75,76,77]. Nevertheless, the PTP-BL PDZ2 analysis relates nonstable conformations (in the classical manner of open/close) but the folding process itself and binding. 

## 5. Plasticity in PDZ Targets: Folding Events upon Binding in PDZ Interactions

There is a wide range of PDZ interactions with known and diverse partners: the canonical interaction with C-terminal tails of protein targets, internal peptide sequences, other PDZ domains, and phospholipid binding [8]. Importantly, in the considered canonical interaction that implies the C-terminus of the protein targets, an arrangement of the ligand between β2 and α2 is established with β-strand type of interactions (see [51]). Thus, there is a coupled event of folding upon binding on the side of the PDZ targets since there is a structural arrangement after or during a binding event. 

The case of the PDZ-PDZ interaction between nNOS and PSD95 [78], which is key in numerous neuronal functions [79,80,81,82], is similar. nNOS-PDZ/PSD95-PDZ2 complex is known to have an interdomain β-sheet arrangement formed by two β-strands from PSD95-PDZ2 and a β-finger in nNOS [83,84] considered as an extra-structure of the canonical PDZ fold. Jemth’s group analyzed the binding kinetics to decipher if the β-finger in nNOS-PDZ comprises an ensemble of conformations previous to the interaction since this extra-structural element is better arranged upon binding. The authors showed that the contacts that consolidate the PDZ/PDZ complex are cooperatively formed after the main transition state. Remarkably, one of the tested mutations altered the kinetic profile of the interaction in such a way that a conformational change, attributed to the β-finger structuration of nNOS-PDZ, was the rate-limiting step [85]. The tuning of the folding energy landscape by a mutation engendered a folding process in nNOS-PDZ coupled to binding. Plausibly, the majority of PDZ interactions may imply a flexibility that is then reflected in their biological role of scaffolding and signaling.

## 6. Conclusions

PDZ domains act as scaffold and signaling proteins, which causes one to wonder why a single domain can interconnect varied proteins and targets. Apart from a plausible different spatiality and timing availability of the targets according to the conditions, domain plasticity may also be a cause of the function versatility in PDZ, which might be adapted for the different targets. Certainly, the ruggedness of the folding landscape in PDZ domains and allosteric phenomena reflect such plasticity, which can be further modulated by post-translational modifications. Remarkably, other protein families, such as calmodulin [86], protein kinases [87] or GPCR (G-protein-coupled receptors) [88], together with IDPs, also comprise a conformational plasticity that allows multifunctionality. 

## Figures and Tables

**Table 1:** A comprehensive list of all the folding experiments performed with PDZ domains.

PDZ Domain	Experiment	Reference
PDZ2 PTP-BL	Kinetics of WT ^1^	[15]
	φ-value analysis and MD ^2^ of WT	[16]
	Kinetics with an induced folding with peptide	[17]
	Kinetics of a circularly permuted variant	[18]
	φ-value analysis and MD of a circularly permuted variant	[19]
	Kinetics of an alternative spliced form	[20]
	φ-value analysis and MD of an alternative spliced form	[21]
PSD95-PDZ1	Kinetics of WT	[22]
PSD95-PDZ2	Kinetics of WT	[22]
	Kinetics of WT^1^ in the presence of Na_2_SO_4_	[23]
	Kinetics of an amide-to-ester mutant	[24]
PSD95-PDZ3	Kinetics of WT and native-state hydrogen exchange	[25]
	Kinetics of WT	[22]
	φ-value analysis and MD	[26]
	Kinetics of a construct lacking α3 helix	[27]
nNOS-PDZ	Kinetics of WT	[22]
D1pPDZ	Kinetics of WT	[28]
	φ-value analysis and MD of WT	[29]
SAP97-PDZ2	Kinetics of WT	[7]
	Kinetics of WT in the presence of Na_2_SO_4_	[23]
	Kinetics of a circularly permuted variant	[30]

^1^ WT: wild-type PDZ domain. In several cases, a Trp residue is included by a point mutation to follow the kinetics spectroscopically. ^2^ MD: molecular dynamics simulations.
